# Case-Control study of Firefighters with documented positive tuberculin skin test results using Quantiferon-TB testing in comparison with Firefighters with negative tuberculin skin test results

**DOI:** 10.1186/1745-6673-1-28

**Published:** 2006-12-19

**Authors:** James L Fleming, Timothy L England, Howard B Wernick, Steven Reinhart, John A Dominguez, Patrick L Kelley, Forrest D Gorter, Victor Papst, Alicia LaDuke

**Affiliations:** 1 Medical Director, Phoenix Fire Department Health Center, Banner Health System, 150 S. 12^th ^Street, Phoenix, AZ 85007, USA; 2 Assistant Medical Director, Phoenix Fire Department Health Center, Banner Health System, 150 S. 12^th ^Street, Phoenix, AZ 85007, USA; 3 Staff Physician, Phoenix Fire Department Health Center, Banner Health System, 150 S. 12^th ^Street, Phoenix, AZ 85007, USA; 4 Phoenix Fire Department Health Center, Banner Health System, 150 S. 12^th ^Street, Phoenix, AZ 85007, USA

## Abstract

**Background:**

Phoenix Firefighters have had abnormally high rates of tuberculin skin test (TBST) results on medical surveillance. The objectives of this study were to evaluate our firefighters using QuantiFERON-TB (QFT), comparing the results to their TBST results.

**Methods:**

Using QFT results obtained during the study, we compared previously positive TBST responders (Cases) to negative responders (Controls). We also compared both groups for QFT results for *Mycobacterium avium *(MA) exposure.

**Results:**

QFT effectively monitored our working population. 12.9% of the 148 cases, and 3.2% of the 220 controls had a positive QFT result. Another 14.8% of cases and 4.5% of controls had conditionally positive QFT results. There was an unusually high rate of MA response on QFT testing in both groups.

**Conclusion:**

Phoenix Firefighters have a higher than expected TBST and QFT results, which cannot be explained by the increased MA rate. The decreased level of QFT positivity in comparison to TBST results may indicate a considerable false positive TBST rate. The QFT offers many advantages as a surveillance method over TBST in exposed worker populations.

## Background

Tuberculosis (TB) has long been a disease that affects humans. In many areas of the world, it remains a major cause of morbidity and mortality. In the United States, effective diagnosis and treatment have reduced disease rates significantly, especially into the 1980s. However, there was a resurgence of TB with several outbreaks among health care populations in the late 1980s [1]. This led to more consistent monitoring and medical management of health care workers, including Occupational Safety and Health Administration proposed regulations for viable monitoring programs [2]. While the proposed standard was rescinded, worker protection requirements were incorporated into OSHA's Respiratory Protection standard [3].

Tuberculin Skin Testing (TBST) using Purified Protein Derivative (PPD) has been the standard for monitoring health care workers and first responders for latent tuberculosis infection (LTBI). However, PPD testing does have limitations. The predictive value of a positive test result is directly influenced by the prevalence of disease in a population [4]. The level of nontuberculous mycobacterial infection rates within the community can affect specificity by increasing the proportion of false positives and thus influencing the positive predictive value [4]. For this reason TBST is considered positive at varying levels of localized reaction, depending on the likelihood of exposure[4]. In addition, the techniques for intradermal injection, and potential variability in interpretation of test results can reduce the effectiveness in using TBST for medical surveillance. Health care workers are classed in the group at increased risk where a TBST response of 10 mm would be considered a positive response. This allows for more individuals to be covered, however, it also leads to a higher incidence of false positive testing [5].

TBST has been used as part of annual medical evaluation of Phoenix Firefighters since 1990. This testing was started due to an increased risk of occupational exposure to *Mycobacterium tuberculosis *as part of medical first response duties (medical response makes up over 80% of firefighter call outs for the Phoenix Fire Department). Because of this potential exposure, firefighters have been considered as exhibiting a positive TBST response whenever they show a 10 mm or greater result, consistent with other health care workers. From 1992–1996, Phoenix Firefighters experienced a much higher than expected positive TBST response. An investigation was performed by the Arizona Department of Health, and no definitive explanation was found to explain why this high level of TBST conversion occurred [6]. There has not been a single case of active TB among this group of firefighters as of the time of this report, although less than 40% of firefighters who had TBST conversion elected to take prophylactic isoniazid therapy. One hypothesis raised in the final report was exposure to *Mycobacterium avium *(MA) causing a false positive response.

In 2001, Cellestis, Inc^@ ^received approval from the FDA for QuantiFERON-TB^@ ^(QFT). QFT is an in-vitro diagnostic test that measures a cell mediated immune response in a sample of human whole blood, and is based on the measurement of Interferon-gamma secreted from stimulated T cells previously exposed to TB [7]. The QFT also measures Interfeon-gamma from MA, as a control measure [7]. In mid 2004, Cellestis, Inc^@ ^fielded a new version of the QFT, called the Quantiferon Gold. QFT-TB Gold uses synthetic peptides based on the amino acid sequences of the TB-specific antigens CFP-10 and ESAT-6, as opposed to QFT-TB using tuberculin as the TB antigen. As this occurred in the middle of our data collection, we elected to continue to use the initial QFT kits.

Use of the QFT may help resolve problems inherent with using TBST as a screening tool. The Centers for Disease Control (CDC) has only provided qualified support for use of the QFT, indicating that any positive QFT result must be verified by TBST confirmation [8]. Just recently, the CDC has given approval for QFT Gold to be used in place of TBST as a surveillance tool in worker populations [9].

The aims of this study are to: 1) compare QFT results to TBST results in a population where a high incidence of positive LTBI results are present; and 2) determine if MA is a confounder in TBST testing among our firefighters.

## Methods

Participants were chosen from among City of Phoenix firefighters. This group was used because of their previous history of TBST positivity rates, and because they represent a healthy worker population, made up of US citizens who are unlikely to have prior immunization to BCG or health conditions that would decrease their immune response. Also, TB skin testing performed a the PFDHC follows a specific testing protocol, by health personnel trained in proper Mantoux intradermal injection techniques, and with objective reading of the skin test results by a trained observer. All positive and questionable skin test readings are referred to a Clinic physician for final assessment. Prior to initiation of this study, IRB approval was obtained from the Banner Health Research Institute.

Participants were categorized as either subjects (individuals who had documented positive TBST responses within the Phoenix Fire Department Health Center [PFDHC] database) or controls (individuals who had documented negative TBST responses). Subjects were identified through review of the PFDHC database. All subjects were sent a letter asking for their participation. Controls were selected from among volunteers who were having their blood drawn as part of their annual medical evaluations.

There were a total of 238 firefighters listed in the PFDHC database who have a documented positive TBST, out of approximately 1500 current firefighters. Of the potential Subjects, 150 (63.0%) volunteered to participate in the study. Control volunteers were obtained from those Phoenix firefighters who have maintained a negative TBST. Size of the Control group was determined by the number of eligible firefighters who volunteered when they presented for their annual medical evaluation during the study collection period. Study collection occurred from February 1 through September 30, 2005, an 8-month period of time. Of possible Controls, 224 (approximately 18%) firefighters volunteered to participate. Four Controls and two Subjects did not meet the eligibility criteria, and were excluded, leaving 148 subjects and 220 controls.

Blood was collected per instructions of the testing laboratory and transported to the lab within the specified period of time. For this study, the Maricopa County Laboratory performed the testing in accordance with manufacturer's methodologies. Results were transmitted in compiled format from the laboratory to the Principal Investigator. Subjects and controls were provided with their individual test results. In addition, the principle investigator, obtained the following information from the Health Center database: Year of Birth, Year of Hire, date of positive TBST (in subjects) or most recent TBST (in controls), and TBST measurement results.

Statistical analyses were performed using Stata version 9.1 (StataCorp, College Station, TX) by a trained statistician from the University of Arizona.

In order to assess the QFT as an alternative diagnostic screening tool in this occupational population of firefighters, TBST was considered the gold standard for comparison, as it was the recommended screening test by the Center's for Disease Control (CDC). Sensitivity, specificity, as well as positive and negative predictive values were calculated.

The nature of the data collected provided for a matched-pair analysis, as each subject has had both a TBST and a QFT. Utilizing the discordant pairs (a matched pair in which the outcomes are different for the members of the pair), McNemar's test was performed to test if there is an association between a positive TBST and a positive QFT response.

Equivalency tests were also performed, using the Kappa statistic (κ), which makes use of concordant pairs (a matched pair in which the outcome is the same for each member of the pair) to test the level of agreement between the two tests, correcting for the proportion of agreement due to chance [10].

Tests for each of the statistics were run with conditionally positive QFT values treated one of three ways: (1) excluded from analyses; (2) recoded as a positive response; and (3) recoded as a negative response to TB infection. Analyses were also run according to the degree of reaction from the TBST.

## Results

This study observed 368 Phoenix firefighters between the years of 1990–2005, of which 346 (94.0%) were male. The average age at the time of hire was 27 years (range 19 – 48 years), while the average age at the time of QFT testing was 43 years (range 21 – 76 years). We confirmed that all subjects were U.S. born citizens, free of diseases suggestive of immune suppression, and with no previous history of BCG usage.

**Figure 1:**
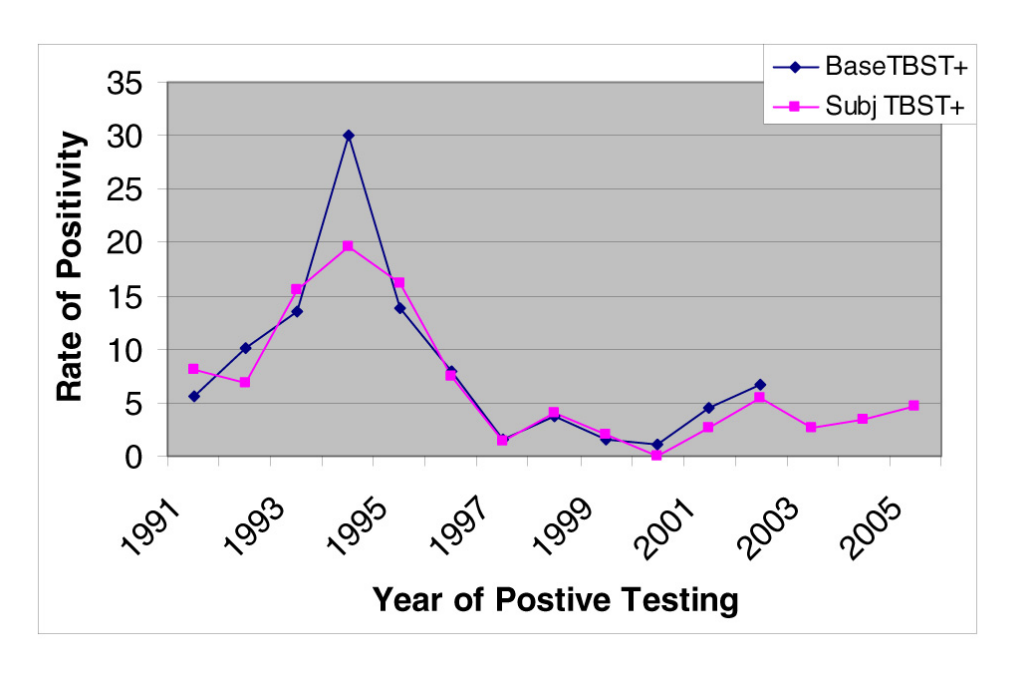
Distribution of positive TBST rates by year of positive response, comparing study subjects to total population distribution.

Of the 148 cases with a positive TBST, 19 (12.8%) resulted in a positive QFT TB response, while 22 subjects (14.8%) resulted in a conditionally positive response. Of the 220 controls (firefighters with no history of a positive TBST), 7 (3.2%) were positive and 10 (4.5%) resulted in conditionally positive responses. Figure 1 shows the comparison of the study cases to the base population, showing a good representation of the population of concern. Table 1 shows the comparison of the QFT results in both the Case and Control groups.

**Table 1:** 2 × 2 Table Comparing TBST Results to QFT Results

	QFT+ (QFT-Cond+)	QFT-
TBST+ (Subjects)	19 (22)	129
TBST- (Controls)	7 (10)	213

Although this study compares two screening tests, the TBST is considered the gold standard for the purpose of this study. As such, depending on how conditionally positive QFT results are treated, sensitivity ranged from 12.8 – 27.7%. Specificity values were much higher, ranging between 92.3 – 96.8%. Positive predictive values ranged between 70.7 – 73.1%, while the negative predictive value ranged from 62.3% to 65.5%. Table 2 displays the results.

**Table 2:** Baseline results

	Conditionally Positive QFT Treatment		
	**Excluded**	**QFT positive**	**QFT Negative**
**Sensitivity (%)**	15.1	27.7	12.8
**Specificity (%)**	96.7	92.3	96.8
**PPV (%)**	73.1	70.7	73.1
**NPV (%)**	65.5	65.5	62.3

**Kappa**			
Agreement (%)	66.1	66.3	63.0
κ statistic	0.1396	0.2218	0.1116
p-value	<0.001	<0.001	<0.001

**McNemar's**			
p-value	<0.001	<0.001	<0.001

For each of the primary analyses, the McNemar's chi-square for matched-pairs was statistically significant. The null hypothesis is therefore rejected, implying that there is a significant difference in how the TBST and QFT classify cases and controls.

As seen in Table 2, the strength of agreement between tests ranged from 0.05 (slight) to 0.22 (fair), based on the arbitrary kappa interpretations from Landis and Koch [9]. In each case, the κ statistic was statistically significant, thus the null hypothesis is rejected and one can conclude that the level of agreement is higher than what is expected by chance.

It has been postulated that a positive response to the TBST may actually be due to a cross reaction with other mycobacterium, to include MA infections, and may result in the misinterpretation of the skin test [6]. The QFT is able to assess MA, as well as TB response. Positive results for MA were found in 125 (34.0%) of the total 368 firefighters, and were equally distributed among cases and controls. For all positive TBST cases, 47 (31.8%) of 148 were positive for MA, while 78 (35.5%) of 220 positive MA responses came from the control group. These results can be seen as a 2 × 2 description in Table 4.

**Table 4:** 2 × 2 Table Comparing TBST Results to QFT MA Results

TBST+ (Subjects)	47	102
TBST- (Controls)	78	142

To evaluate if MA was potentially responsible for the poor level of agreement between the two tests, negative QFT results or conditionally positive QFT results that were positive for MA were recoded as positive for QFT, and all tests were rerun. Results can be seen in Table 3 showing a higher sensitivity, lower specificity and PPV, and similar NPV, when this recoding is performed. The strength of agreement was lower than what was originally seen, prior to this recoding, suggesting that miscategorization as MA-positive is not responsible for the poor level of agreement between the two tests.

**Table 3:** Results with +MA recoded as +QFT

	Conditionally Positive QFT Treatment		
	**Excluded**	**QFT positive**	**QFT Negative**
**Sensitivity (%)**	52.4	59.5	44.6
**Specificity (%)**	59.5	56.8	61.4
**PPV (%)**	43.7	48.1	43.7
**NPV (%)**	67.6	67.6	62.2

**Kappa**			
Agreement (%)	56.9	57.9	54.6
κ statistic	0.1145	0.1567	0.0594
p-value	0.0168	0.0011	0.1273

**McNemar's**			
p-value	0.0379	0.0049	0.8164

All statistical analyses were again run according to the average size of reaction to the TBST in millimeters (mm). Size categories ranged between 10 – 20 mm in intervals of 2 mm, as well as those less than 10 mm and greater than 20 mm. Many of the results were inconclusive as the numbers of observations in some instances were too low for analysis. Categorization was then reordered into quartiles based on an equal distribution of observations. Results did not differ from what has been recorded above.

**Figure 2:**
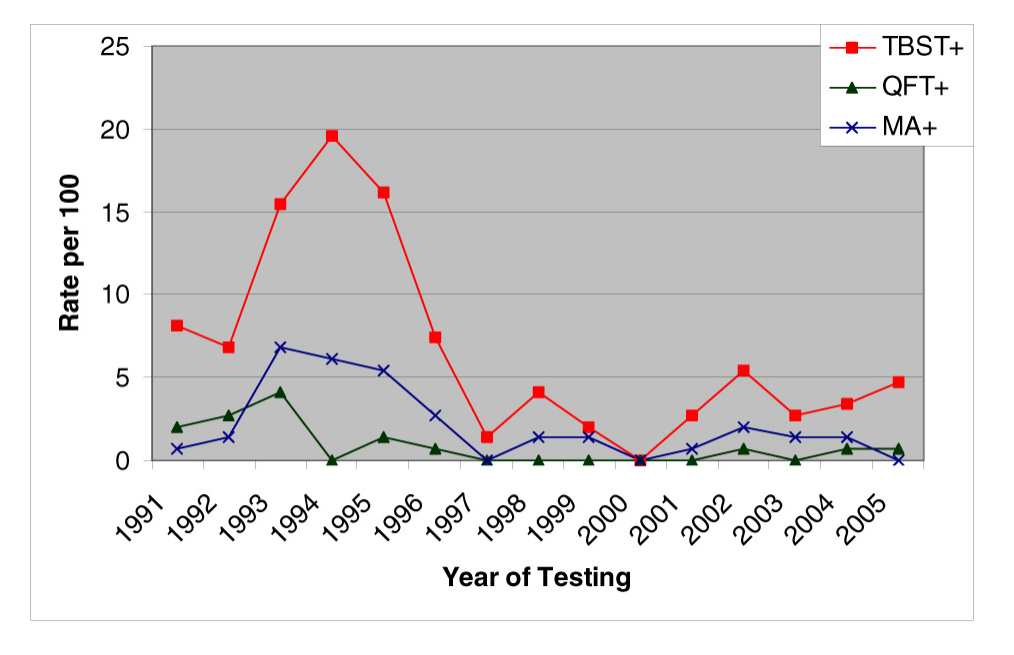
Rate per 100 for TBST positivity and QFT positivity by year positive TBST finding.

To assess whether time since TBST testing in comparison to QFT testing was an issue, we compared the rate of positivity by year of TBST positivity (See figure 2). We noted that while MA positivity had a mild upswing correlating with TBST responses, TB positivity by QFT does not appear to be affected. While not a direct part of the study, we noted that a subset of the subject cases (35) have had recent TBST's (within the last 3 years) as part of their ongoing medical evaluations. Only 4 of the cases had a positive response on the repeat testing. 2 of those 4 had a positive QFT response with one showing a positive MA response. Of the 31 who have had recent negative TBST response, 1 had a positive QFT response for TB, with 3 others having conditionally positive response, and 13 having a positive MA response.

## Discussion

We found that there was fair to low agreement between TBST and QFT. However, it is not clear which is a "better" test. There is an inherent problem with comparing a new screening test to the one that is currently available, in the absence of a gold standard, other than active TB. This limits the ability to decisively state that one particular test results in a more favorable outcome. The time difference between TBST response and when QFT testing is performed may also impact on the comparability of the two tests. The realization that 31 of 35 cases with previous TBST positive response subsequently tested negative lends argument that a fair number of the cases may not be infected with TB. All that can be concluded is that the tests do differ. To determine if one test better screens for TB, results from a confirmation test (e.g. chest x-ray, acid-fast bacilli smears from sputum, or isolation of My*cobacterium tuberculosis *complex on culture) would have to be known. As none of our subjects have developed active disease, comparison of the QFT to a confirmation procedure is not available. It is our intent to continue to follow our firefighters in ongoing surveillance.

These limitations aside, this study does show that the QFT does result in a significantly lower rate of positivity to LTBI than TBST. The rate of positivity, regardless if from TBST or QFT is high for a healthy work force (TBST positivity over 15 year period is 15.9 per hundred firefighters and QFT Positivity is 7.1 per 100 for the 8 month study period). Also, the elevated trend in TBST test results in the 1992–1996 period is not supported by the QFT results, as demonstrated in Figure 2. This lends credence to the initial assessment of the Arizona Department of Health that the TBST results were false positives^6^.

The lower rate of positivity using QFT, even including conditionally positive results, indicates that there should be less of an issue with false positive responses using QFT, even though comparison with TBST can only tell that the rates are significantly different. Continued monitoring of our positive responding firefighters for evidence of active TB may help resolve this question. We intend to re-start TB skin testing on individuals who have tested negative on QFT, even if previously skin test positive. This may provide additional insight in comparing these two tests. Also, studies on other healthy population groups may help resolve some of these questions.

The prevalence of MA among subjects and controls suggests that miscategorization as MA-positive is not a confounder in the subjects. This is supported in Table 2, suggesting that there must be some explanation, other than MA infection, to the high level of TBST response in firefighters, especially during 1992–1996. Other infections could have caused the increased rate of TBST positivity at that time, or there could have been improper procedures of testing during that period of time.

There was a high rate of MA positivity in our test population, both subjects and controls. This could indicate that MA is highly prevalent in our community, that our firefighters are more likely to become infected with MA than other groups within our population, or that there was a high false positivity not truly reflecting actual MA infections. The health impact of MA infectivity on this healthy work group is not known, although no apparent health effects have been noted. Further studies to compare our firefighters to the local population and/or versus other workgroups are recommended.

## Conclusion

Firefighters of the Phoenix Fire Department have a higher than expected rate of positive TB response^6^. This increased rate cannot be explained by an increased MA exposure. The decreased level of positive response to QFT suggests, along with the lack of any active TB cases among our subjects, that there has been a high false positive TBST rate.

## Competing interests

The author(s) declare that they have no competing interests.

## Authors' contributions

All authors participated in the proposal and preparation of the study. They also actively participated in the data collection process. JF performed the main writing of the proposal, IRB approval, data collection, data analysis, and writing the final paper. All authors actively participated in reviewing/editing of the final paper for submission.
